# In Vitro Comparison of Internal and Marginal Adaptation between Printed and Milled Onlays

**DOI:** 10.3390/ma16216962

**Published:** 2023-10-30

**Authors:** Oriol Cantó-Navés, Kyra Michels, Oscar Figueras-Alvarez, Sandra Fernández-Villar, Josep Cabratosa-Termes, Miguel Roig

**Affiliations:** 1 Department of Restorative Dentistry, Universitat Internacional de Catalunya, 08195 Sant Cugat del Vallès, Barcelona, Spain; ocanto@uic.es (O.C.-N.); sandrafernandez@uic.es (S.F.-V.); cabratosa@uic.es (J.C.-T.); mroig@uic.es (M.R.); 2 Universitat Internacional de Catalunya, 08195 Sant Cugat del Vallès, Barcelona, Spain; kmichels1@uic.es

**Keywords:** 3D-printed onlays, milled onlays, indirect restoration, resin, graphene

## Abstract

Statement of problem: Nowadays, milling is still the gold standard for fabricating indirect restorations, but to overcome its disadvantages, there are alternatives, such as 3D printing. Purpose: This study aimed to compare the gaps between the prepared tooth and milled and printed onlays fabricated with the same CAD design. It also aimed to determine the gap reproducibility across onlays fabricated by 3D printing and milling. Methods: A resin tooth was prepared for an onlay. After scanning the preparation, an onlay was designed with proprietary dental software. Next, 22 onlays were milled in a graphene-reinforced PMMA disc (Group 1), and 22 onlays were 3D-printed with a hybrid composite material (Group 2). After that, all fabricated restorations were scanned and superimposed on the scanned prepared resin tooth. Subsequently, a specific software was used to measure the margin, central, and intaglio-located gap between the milled or printed restoration and the preparation. Finally, measurements were compared with a multifactor analysis of variance. Results: The results demonstrated that printed onlays (Group 2) adapted better to the prepared tooth than the milled ones (Group 1) (*p* < 0.05). The comparison of standard deviations showed the better gap reproducibility of printed onlays (*p* < 0.05). Conclusion: This study concluded that the printed onlays adapted significantly better to the prepared tooth than the milled onlays. Printed onlays also showed significantly better gap reproducibility.

## 1. Introduction

Loss of dental tissue is a common consequence of caries, erosion, abrasion, wear, fracture, or a combination of those factors. Nowadays, highly developed restoration materials and adhesive dentistry allow preparation and placing direct or indirect partial restorations instead of full crowns, which are more invasive [1,2,3].

There are no significant differences in longevity between direct and indirect restorations, so both might be utilized for replacing missing tooth structures. However, direct restorations are more prone to suffer from future filtration and generate more stress in the cavity’s remaining walls due to the shrinkage of polymerization [3]. Indirect restorations are more useful for replacing more significant amounts of missing tooth structure in more significant cavities because the shrinkage of polymerization-generated stress only appears in the cement layer and, therefore, is minimized in the preparation cavity walls [2]. Indirect partial restorations need tooth preparations that provide a minimum thickness of 1.5 to 2 mm, depending on the future restoration material [1,4].

The digital dentistry workflow includes quick and precise intraoral scanning, the digital on-screen design of the restoration, and design production using milling or 3D printing [4,5,6,7]. Even though there seems to be a better adaptation of printed restorations [8,9,10,11], the subtractive method is still the gold standard for the CAM process, in which the production accuracy and precision of the previously digitally created restoration design are determined by the number of working axes of the computer numerical control machine and the size of the milling burs. The current techniques allow the milling of almost all types of material, from the softest ones, such as waxes, to the hardest ones, such as metal [8]. The most interesting materials regarding indirect partial restoration production are dental ceramics, hybrid materials, and composites. Dental ceramics display better optical properties and mechanical characteristics but are more prone to fractures and chipping during milling and after intraoral cementation. They are also more challenging to repair intraorally than other materials. Hybrid composite materials are an attractive alternative to dental ceramics because they offer good optical and mechanical properties and are easy to fabricate, cement, and repair [3,12]. Regarding new materials in digital dentistry, graphene is highlighted for clinical use in various fields due to its excellent properties [13,14,15,16,17,18,19,20]. Although its use in indirect restorations still shows mixed reports, it might be beneficial to investigate its use as a permanent prosthesis [15,16,18,19,20]. Graphene is a 2D single layer of hybridized carbon atoms and various graphene-family nanomaterials with different surface properties, numbers of layers, and sizes [5,13]. Two leading graphene derivatives are graphene oxide and reduced graphene oxide. Graphene oxide is of interest in dentistry because of its mechanical properties and excellent biocompatibility [7,17]. Incorporating graphene into PMMA improves mechanical properties and decreases the degree of contraction during polymerization [15,16,18,19,20]. Furthermore, graphene shows good biocompatibility with soft and hard tissues and reduces the effects of free radicals of the PMMA. It also has certain antimicrobial adhesion effects due to its hydrophilicity [19,20]. Because of these advantages, graphene-reinforced PMMA is a promising material for indirect restorations that can be easily milled.

Nevertheless, milling has some limitations, so micro-cracks may appear in the material during the milling process, and accessing to the smallest hollow areas or slight undercuts may be challenging [8,21].

CAM additive methods are promising to overcome some of the limitations of milling. They are economical in terms of hardware investment and overall production costs. They also avoid the waste of the non-used material of the disk or the block after milling. Various technologies exist among the additive production methods, including powder bed fusion (PBF), fused deposition modeling (FDM), and light curing. Ceramic particles can be incorporated into the resin matrix of a hybrid composite to improve its mechanical properties [22,23,24,25]. A light-curing hybrid material reinforced ceramically was developed to manufacture single-tooth restorations with 3D printers (Permanent Crown Resin; Bego GmbH). After printing, the restoration must be cleaned with isopropyl alcohol and air-abrasion, post-polymerized, and improved esthetically with composite-resin stains. These printed restorations are proven to be mechanically and chemically stable, to have appropriate biocompatibility, not to release harmful substances, to show a smooth surface that avoids the adherence of oral cavity bacteria, and to be esthetically pleasant [25,26].

The adaptation of indirect restorations can be assessed via the measurement of the gap, which is the distance between the prepared tooth’s walls and the restoration’s internal surface. The size of the gap can highly influence the longevity of the restoration, the decoloration and degradation of the luting agent, the bacterial leakage, and the ability of the restoration to withstand loading [7,27]. This study aimed to compare the gaps between the prepared tooth and graphene-reinforced PMMA milled onlays and hybrid composite 3D-printed onlays fabricated with the same CAD design. It also aimed to assess the gap reproducibility of onlay indirect restorations made with an additive (3D printer) and a subtractive method (5-axis milling).

## 2. Materials and Methods

**Figure 1:**
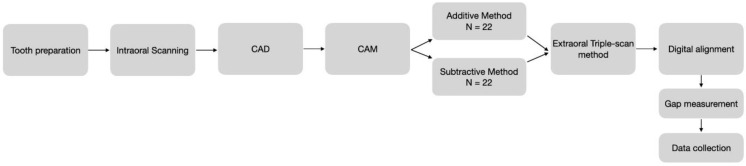
Workflow of study.

This study was conducted in an in vitro environment following the workflow represented in Figure 1. Ethical approval was registered as TFG-20223-A94 from the Comitè d’Ètica de Recerca-UIC on 21 December 2022.

### 2.1. Sample Preparation

**Figure 2:**
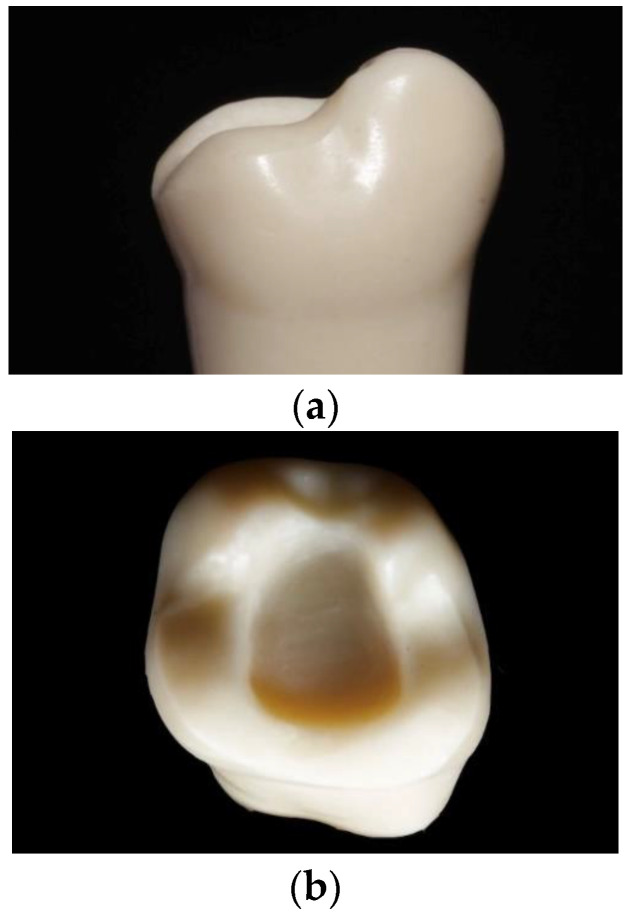
Prepared tooth: lingual (**a**) and occlusal aspect (**b**).

**Figure 3:**
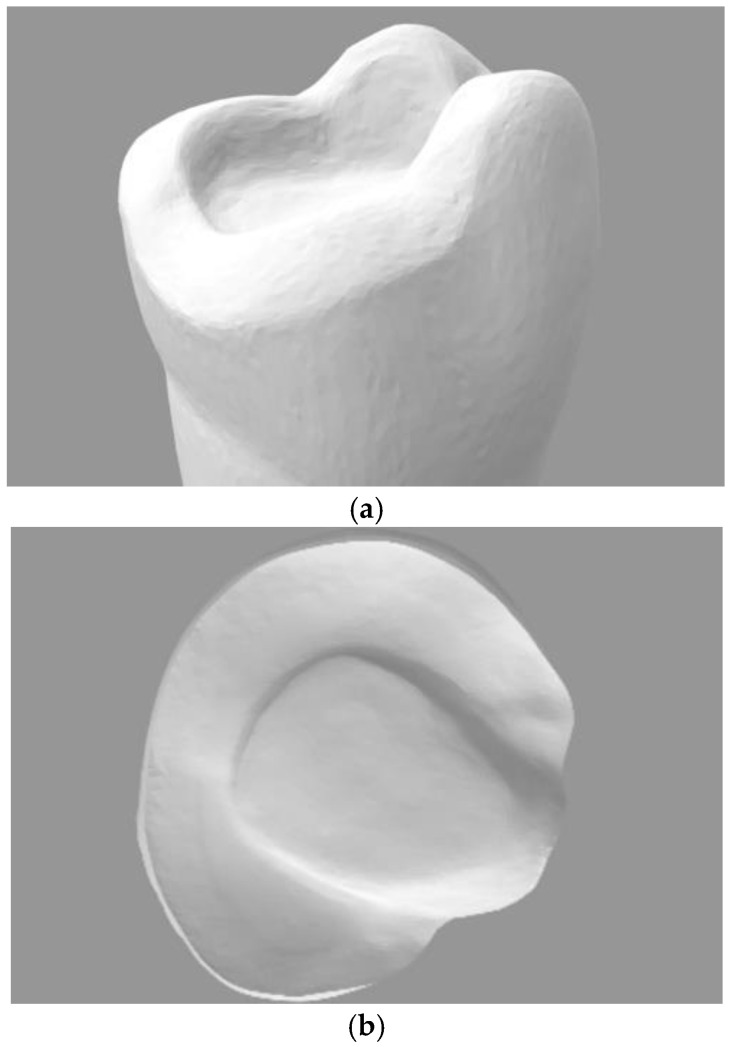
STL file of the prepared tooth. Lingual (**a**) and occlusal (**b**) aspect.

An onlay preparation on an upper-right first resin molar was performed with diamond burs (Conical and Round Diamond Burs 016; Dentsply Sirona, Charlotte, NC, USA) and polished with discs (Enhance Multi Refill Polishing Discs; Dentsply Sirona) (Figure 2a,b). Distal cusps were maintained, the heights of mesial cusps were reduced by 3 mm, and a wall thickness of 2–3 mm was kept. The outer margin of the preparation followed the design of a chamfer, and a central box of 3 mm depth with an insertion angle of 10° was created in order to be able to look at the future internal adaptation in more difficult zones. Then, the prepared molar was scanned using an intraoral scanner (Trios3 Move^+^, 3Shape A/S). The resulting STL file (Figure 3a,b) was uploaded into proprietary dental CAD software (Exocad Galway, Exocad GmbH, Darmstadt, Germany) for designing an onlay with the natural anatomy of the upper first molar adapted to the prepared margin.

### 2.2. Sample Manufacturing

The STL file of the designed onlay was imported into the milling machine and 3D-printer CAM software (Preform 3.32.0, Formlabs, Somerville, MA, USA) for production. For the subtractive manufacturing process, a graphene-reinforced PMMA was used. The additive method was conducted using a hybrid composite, which is currently an auspicious material ideal for indirect restorations. 

**Figure 4:**
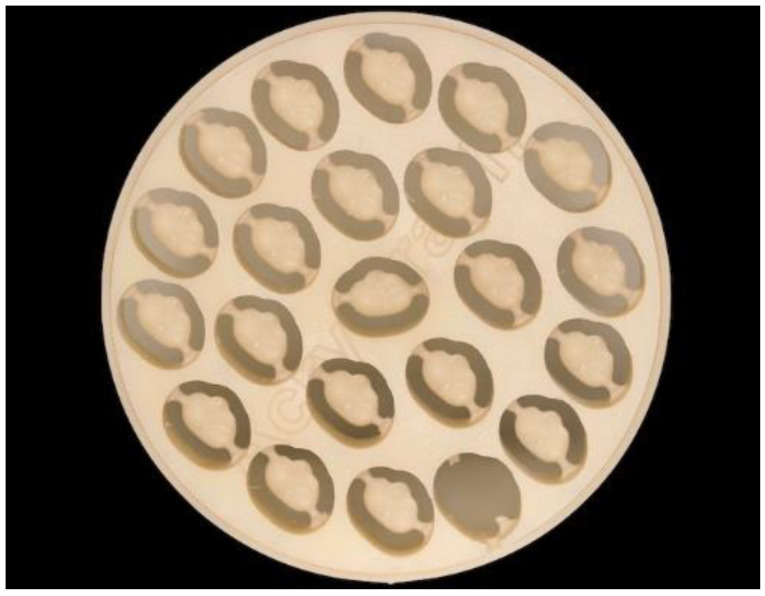
Milled onlays (Group 1).

Due to the maximum capacity of one disc, twenty-two onlays (Group 1) were dry-milled from a graphene-reinforced PMMA block (Acrylgraph; Nuprodent SL; Table 1 with a 5-axis milling machine (K5^+^; VHF cam-facture AG) (Figure 4). Burs for indirect restoration fabrication were used. The milled onlays were removed from the disc by cutting the connections with a bur (Diambconflat end FG M 014; Dentsply Sirona).

**Figure 5:**
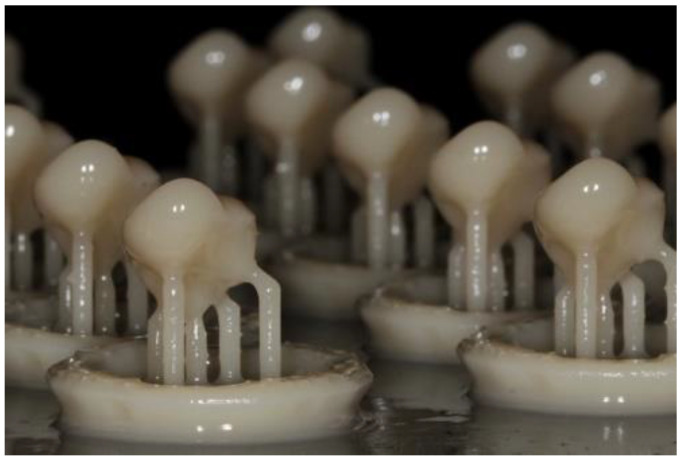
Printed onlays (Group 2).

Following the sample size of Group 1, another 22 onlays (Group 2) were SLA 3D-printed (Formlabs Form 3^+^; Formlabs GmbH, Berlin, Germany) using hybrid composite material (Permanent Crown Resin A2; Bego GmbH, Bremen, Germany; Table 1) (Figure 5). The 3D printer parameters were set at a 50-micron layering dimension with a laser power of 250 mW. After the printing, the restorations were cleaned with isopropyl alcohol for 3 min and air-dried before the post-polymerization process, which consisted of two cycles of 20 min at 60 °C temperature, as recommended by the manufacturer. The excesses were finally removed with a fine bur (Diambconflat end FGM014; Dentsply Sirona, Mecklenburg County, NC, USA).

The resulting onlays were not polished or finished. Each onlay of each group was numbered from 1 to 22 to be followed correctly throughout the process. 

### 2.3. Digitalization of Samples

A strict scanning protocol to digitalize (1) the original tooth before preparing the onlay, (2) the prepared resin tooth, (3) all printed and milled onlays, and (4) all printed and milled onlays on the prepared tooth was performed using an extraoral scanner (Medit T500; Medit Corp.). Printed and milled onlays were reversibly placed onto a small stick on its lingual side to facilitate the scanning. When scanning the onlay on the prepared tooth, a small amount of silicone (Zetalabor; Zhermack GmbH, Marl, Germany) was placed on the lingual aspect of the prepared tooth to slightly fix every onlay on it during the scanning process. The part where the silicone was placed was further cut out from the STL in order to prevent wrong measurements. Forty-six scans were made (44 onlays and the unprepared and prepared tooth). All scans were saved as STL files and named according to the onlay numbers and groups. 

**Figure 6:**
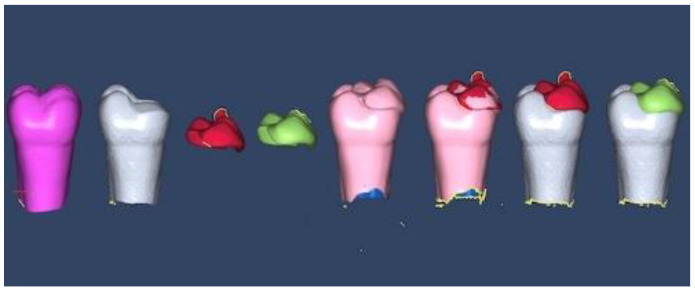
Sequence of aligning the STLs.

The alignments of (1) the original tooth and the prepared tooth, (2) the original and prepared tooth and the onlay, and (3) the onlay and onlay on the prepared tooth were performed using dedicated software (Version 7.4.5, Open Technologies Software; OpenTech3D SRL, Rezzato, Italy) to further measure the gap between the fabricated onlay and the prepared tooth. The gap was assessed by measuring the adaptations between the onlays and prepared tooth scans. Figure 6 shows the alignment protocol and the possibility of placing each onlay separately onto the prepared tooth to measure the gap between them (Figure 6).

### 2.4. Evaluation of Adaptation

**Figure 7:**
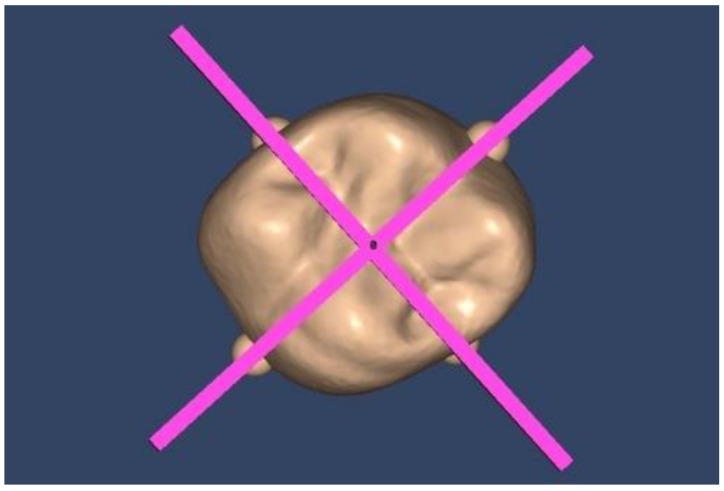
Vestibular–palatal and mesial–distal cut planes through four spheres.

**Figure 8:**
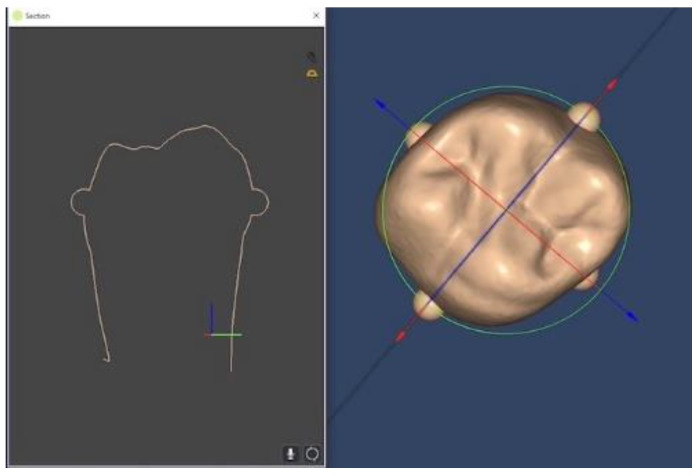
Vestibular–lingual cut plane.

**Figure 9:**
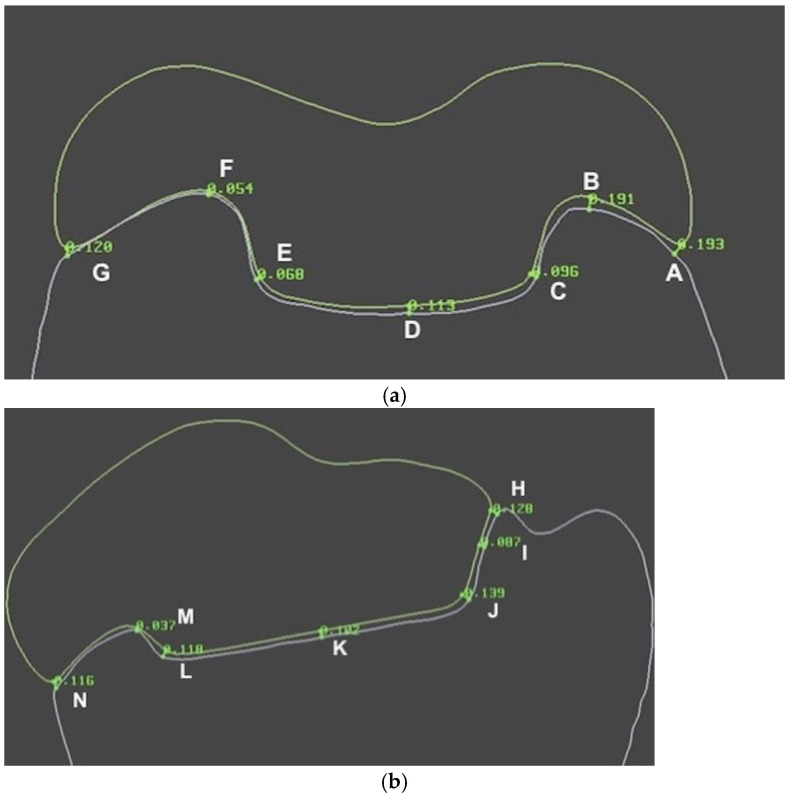
Examples of predetermined points A–G (**a**) and H–N (**b**).

Adaptation was evaluated by measuring the space between each onlay and the prepared tooth at predefined points at the margin and the internal space using proprietary software (Limaguide 1.9.1; Limaguide SL, Barcelona, Spain). The STL files of the original tooth, the prepared tooth, and all 44 onlays were imported into the software. Then, two perpendicular boxes intersecting at the middle of the tooth were established on the original tooth. Furthermore, four spheres were fused to a duplicate of the original tooth scan at the intersection of the outer surface of the original tooth and the previously designed boxes (Figure 7). When measuring the different samples, the spheres established where to place the two cut planes, which were positioned at vestibular–lingual and mesial–distal planes of the tooth through the center of the four spheres. On the vestibular–lingual plane (Figure 8), the prepared tooth replaced the original tooth without altering the cut plane position. Then, each onlay was positioned individually on the prepared tooth scan. Seven predetermined points, named from A to G (Figure 9), were set on the vestibular–lingual plane: two at the margin, four at the inner area, and one at the center of the preparation. After that, all onlays were evaluated the same way on the mesial–distal plane. Seven points, named from H to N, were also set in the same way on the mesial–distal plane: two at the margin, four at the inner area, and one at the center of the preparation (Figure 9a,b).

Gap measurements were made for each separate onlay at the predetermined points of the two cut planes (A–G and H–N) with the measuring tool on Limaguide 1.9.1 software. The measurements were then uploaded to a spreadsheet (Microsoft Excel; Microsoft, Redmond, WA, USA). Means and standard deviations of gap measurements corresponding to the marginal adaptation (points A, G, H, and N), the inner adaptation (points B, C, E, F, I, J, L, and M), and the central adaptation (points D and K) of every onlay were calculated. The resulting means and standard deviations of all samples were subsequently evaluated statistically. 

### 2.5. Statistical Analysis 

After assuring the normal distribution and the homoscedasticity of collected data, multifactor analysis of variance (ANOVA) and Fisher’s least significance difference (LSD) posthoc tests were used to compare milled (Group 1) and printed onlays (Group 2) at the three different measurement spots (margin, inner, and center) using a dedicated statistics package software (Statgraphics Centurion X; Statgraphics Tehnologies Inc., Plains, VA, USA). Statistical significance was set at *p* < 0.05 with a confidence interval of 95%. 

## 3. Results

**Figure 10:**
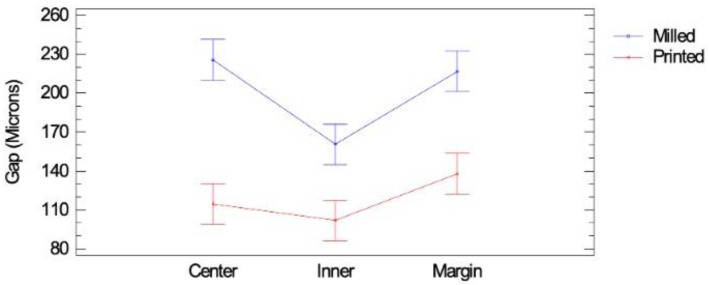
Means of measured gaps of milled and printed onlays at different locations: interaction plot with LSD intervals. In printed onlays, in general, the gaps are smaller than in printed onlays. The biggest discrepancy between the two models is found at the center point, and the smaller discrepancy between both models is found in the inner locations.

The gaps between the prepared tooth and 44 onlays, 22 milled (Group 1) and 22 milled (Group 2), were compared using a multifactor analysis of variance at three spots: the margin, the inner part, and the central part (Table 2, Figure 10). The calculated means and standard deviations for the measured gap on each type of onlay at the parts of the adaptation gap, together with the statistical significances, are shown in Table 3. 

Printed onlays (Group 1) fit significantly better than the milled onlays (Group 2) at the marginal, inner, and central parts (*p* < 0.05) (Table 3, Figure 10). The fit at the inner part of the gap measured in the milled onlays was worse than in the marginal and central parts (*p* < 0.05).

**Figure 11:**
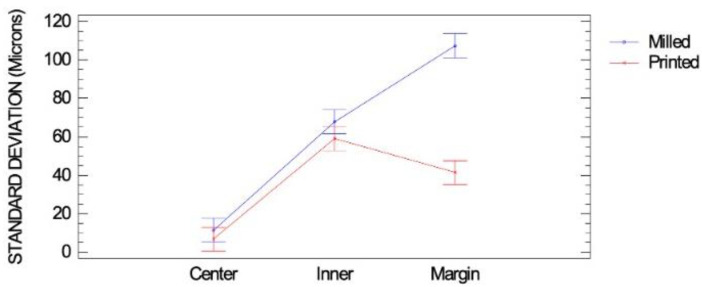
Standard deviations of measured gaps of milled and printed onlays at different locations: interaction plot with LSD intervals. The standard deviations are very similar in both materials at the center and inner locations while, in the marginal locations, there is a great discrepancy between the two materials, with the largest standard deviation being present for the milled onlays and a smaller standard deviation in the printed ones.

It was noticed that the gap reproducibility, determined using the standard deviation of the measured gaps, was very similar for the milled and printed onlays at the central and inner parts. However, a lower gap reproducibility was assessed at the marginal part of the milled onlays compared to the printed onlays, which implied less predictability in the obtained gap in the milled onlays compared to the printed onlays (Figure 11). The detected gap reproducibility at the central part of the onlays was significantly lower than at the inner and marginal parts in both types of onlays, which implied, in both milled and printed onlays, more predictability in the obtained gap at the central part than at the margin and inner part.

## 4. Discussion

The adaptation of the indirect restoration to the prepared tooth plays a vital role in longevity. A significant marginal or internal discrepancy may lead to cement dissolution, microleakage, secondary caries, or restoration fracture. Therefore, obtaining the best adaptation of indirect restoration–preparation must be aimed at in order to prevent secondary complications [1,2,3,21]. Regarding the ideal and clinically acceptable adaptation, most researchers report that the clinically acceptable gap should be between 100 and 150 microns, while other authors state a possible range between 110 and 200 microns [27]. 

This study used graphene-reinforced PMMA milled onlays and hybrid composite 3D-printed onlays in order to assess the adaptation of subtractive and additive CAM methods. These two innovative materials were chosen because of their physical properties and clinical indications as indirect unitary restorations. This paper demonstrated that printed onlays adapted better to the prepared tooth than the milled ones and had better gap reproducibility. The marginal and internal adaptation were assessed and evaluated using a triple scan protocol, which is a valid and nondestructive method [8]. Other alternatives to measure adaptation are described in the literature, such as visual examination, with or without magnification, tactile probing, or the silicone replica technique [9,21,28]. Nevertheless, the digital measuring approach may improve accuracy, reproducibility, and the in-depth performance analysis of the CAD–CAM systems into a fully digital workflow, overcoming the disadvantages of the analogue techniques. 

Even though care was taken to avoid sharp areas in the design, the lower adaptation of the milled onlays might be attributed to the sharp design of the prepared tooth, which could lead to difficulties during the milling process because of the burs that cannot assess concave areas. Although the used 3D printer was not the newest on the market, the adaptation was very good and clinically acceptable. It might be presumed that the fit could be even more precise with a smaller layering dimension (30 microns). 

A scoping review of the marginal and internal accuracy of milled lithium disilicate onlay restorations summarized a marginal gap of onlays ranging from 0.041 to 0.086 mm and an internal gap ranging from 0.092 to 0.096 mm, which is lower than the values found in this study, where the most significant differences at the measured gaps according to the location were measured in the marginal areas of both the printed and the milled onlays (Figure 10). Printed onlays adjusted much better at the marginal spot than the milled onlays (Figure 11), probably because of the needed milling strategy due to the roundness of the margin when resin restorations are to be performed. However, the scoping review condensed many results from various studies using different methodologies, making direct comparison difficult [29]. Different software with different settings was used in each study, which made comparison challenging. Exocad Galway proprietary software with the recommended settings for onlay restorations was used in our study. 

Haddadi et al. reported, in their in vitro research, marginal gaps in the milled and printed crowns made of hybrid composite on extracted teeth of 0.09 mm and 0.07 mm, respectively [9]. Inherent deficiencies that affect the reliability of the results have been reported concerning the replica technique used in their research, making a direct comparison with our in vitro study difficult [30]. Bae et al. also reported a higher accuracy when fabricating the inlays using the printing technique (SLA) than that found when using subtractive methods. A similar scanning protocol and alignment of the resulting STLs to determine the accuracy were used [8]. Karasan et al. also demonstrated a better internal fit with printed fixed dental prostheses than with milled ones and the higher predictability and repeatability of restoration manufacturing when printing [10]. Kakinuma et al. also found that 3D-printed resin-composite crowns showed a better marginal fit than milled ones [11]. They assessed the marginal and internal fit by sectioning the crowns and inspecting them under a laser microscope. Our research samples were also sectioned, but digitally, with the plane cuts being made across the aligned meshes of the prepared abutment and the restoration. Lerner et al. reported no remarkable differences in marginal fit between milled and printed zirconia crowns [21]. However, the marginal adaptation was assessed by using visual analysis and a periodontal probe on a split cast model, which provided more subjective and less accurate results than the digital measuring results of the paper.

On the contrary, Kim et al. reported the best adaptation results for metal fixed dental prostheses made using the subtractive method compared to additive (selective laser sintering) and traditional (lost wax and casting) methods. The injected light body silicone material placed into the cement space was scanned and measured with the appropriate software, so the replica technique and the digital measuring method were combined to assess the adaptation of the fixed dental prosthesis [28]. The methodology and the materials used make the comparison of their results with ours difficult.

Currently, there seems to be a better adaptation of printed restorations [8,9,10,11]. However, no in vivo studies with those printed materials are still available, so it may be difficult to approve their clinical applicability. 

This study has limitations, including the studied material choice and the limited number of digitally measured points at the marginal and internal gaps. Differences were found in comparing graphene-reinforced PMMA and hybrid composite 3D-printed, but caution must be taken when interpreting the results with other materials. All measuring points were the same for all samples because of the protocol followed, wherein using four spheres made the cut plane position reproducible. However, measurements were made only at determinate spots. This could be improved by measuring the existing area between the restoration and the prepared abutment. 

## 5. Conclusions

Within the limitations of the present in vitro study, the following conclusions can be drawn:

1. The printed onlays adapt significantly better to the prepared tooth than milled onlays.
2. A significantly higher gap reproducibility within the group of the printed onlays was demonstrated.
3. Further research is needed to determine intraorally printed restorations’ longevity and long-term behavior.

## Figures and Tables

**Table 1:** Materials.

Product Name	Manufacturer	City/State/Country	Shade	Composition	Lot Number
Permanent Crown Resin	Bego	Bremen, Germany	A2	Esterification products of 4,4’-isopropylidene diphenol (ethoxylated), 2-methylprop-2-enoic acid, silanized dental glass, methyl benzoyl formate, and diphenyl (2,4,6-trimethylbenzoyl) phosphine oxide. Total proportion of inorganic fillers (particle size 0.7 μm) is 30–50% by mass	600,926
Acrylgraph	Nuprodent	Soneja, Spain	A3	Poly(methyl methacrylate)+ Graphene	3 February 2020

**Table 2:** Analysis of variance for measured gaps of two types on onlays (milled and printed) at different locations (margin, inner, and central) and their interaction. All data are measured in microns.

Source	Sum of Squares	Df	Mean Square	F-Ratio	*p*-Value
MAIN EFFECTS					
A: Type	227,327	1	227,327	82.01	0.0000
B: Location	54,414.4	2	27,207.2	9.82	0.0001
INTERACTIONS					
AB	15,310.6	2	7655.28	2.76	0.0670
RESIDUAL	349,251	126	2771.83		
TOTAL (CORRECTED)	646,303	131			

**Table 3:** Means ± standard deviations of gap between milled and printed onlays at different studied locations. p* column indicates ANOVA (LSD results < 0.05). All data are measured in microns.

	1. Marginal GAP	2. Inner GAP	3. Central GAP	p*
Milled (G1)	216.90 ± 107.45	160.57 ± 67.72	225.73 ± 11.31	<0.05 (1–2, 2–3)
Printed (G2)	137.86 ± 41.33	101.74 ± 58.98	114.60 ± 6.62	>0.05
p*	<0.05	<0.05	<0.05	

## Data Availability

Not applicable.
